# Association between socioeconomic context and getting preventive screenings among Brazilian women aged 50 years and older

**DOI:** 10.1590/1980-549720260051

**Published:** 2026-09-28

**Authors:** Poliana Fialho de Carvalho, Luciana de Souza Braga, Sérgio Viana Peixoto, Waleska Teixeira Caiaffa, Maria Fernanda de Lima-Costa, Juliana Vaz de Melo Mambrini

**Affiliations:** I Instituto René Rachou - Fiocruz Minas, Belo Horizonte (MG), Brazil.; II Universidade Federal de Minas Gerais, Belo Horizonte (MG), Brazil.

**Keywords:** Health services, Women’s health, Healthcare disparities, Cross-sectional studies

## Abstract

**Objective:**

To evaluate the association between contextual socioeconomic conditions and indicators of health service use among women aged 50 years or older, according to source of care (Brazilian Unified Health System-SUS vs. private health insurance).

**Methods:**

A cross-sectional study was conducted using baseline data from the Brazilian Longitudinal Study of Aging (2015-2016), a nationally representative survey of the population aged 50 years and older. The following indicators of health service use were analyzed: mammogram, Pap smear, measurements of cholesterol, blood glucose, and blood pressure. Contextual socioeconomic condition was assessed using the Brazilian Deprivation Index (Índice Brasileiro de Privação - IBP). Crude and adjusted multilevel logistic models were fitted after significant between-census-tract variability was confirmed.

**Results:**

The sample included 5,314 women, 54.2% of whom were aged 60 years or older. A socioeconomic gradient was observed in blood glucose and cholesterol testing, with lower odds of undergoing these tests among women living in areas of greater deprivation. This pattern was observed among both SUS users and private health insurance beneficiaries (blood glucose: OR 0.82, 95%CI 0.69-0.96 and OR 0.91, 95%CI 0.87-0.96; cholesterol: OR 0.71, 95%CI 0.59-0.87 and OR 0.89, 95%CI 0.85-0.95, respectively).

**Conclusion:**

The findings highlight socioeconomic inequities in cholesterol and blood glucose testing among women aged 50 years or older, particularly among SUS users living in areas with higher levels of deprivation. These results reinforce the need for public policies aimed at reducing socioeconomic inequities in healthcare utilization among women.

## INTRODUCTION

Despite substantial advances, social inequalities in health persist in Brazil^1^. Studies have shown that these inequalities extend to the use of preventive health services. Individuals with higher income and educational attainment, those living in urban areas and more developed regions, and those with private health insurance are more likely to use these services^2^ ^,^ ^3^ ^,^ ^4^.

Such inequalities affect the occurrence and management of non-communicable diseases (NCDs), which represent a major public health concern^5^ ^,^ ^6^ ^,^ ^7^. NCDs have a high incidence and are the leading cause of morbidity and mortality globally, accounting for 38 million deaths annually, 75% of which occur in low- and middle-income countries^7^ ^,^ ^8^. In Brazil, NCDs accounted for 54.7% of deaths in 2019^5^. Among women, the two leading causes of death were diseases of the circulatory system and neoplasms^9^.

Among neoplasms, breast cancer is the leading cause of cancer-related death in women, while cervical cancer ranks third. Women who are married, highly educated, and have private health insurance are more likely to have undergone mammography and Pap smear testing, which are screening tests for these cancers^10^ ^,^ ^11^ ^,^ ^12^ ^,^ ^13^.

In Brazil, the creation of the Family Health Strategy has positively influenced various health indicators, including those related to NCDs such as reductions in hospitalizations for primary care-sensitive conditions and cardiovascular mortality, making it a key public policy for mitigating the consequences of health inequalities^14^ ^,^ ^15^ ^,^ ^16^. However, the country remains marked by social inequalities regarding access to and use of health services^17^ ^,^ ^18^.

In addition to individual socioeconomic factors, studies indicate that the socioeconomic context plays an important and independent role, highlighting that health disparities are also determined by macroeconomic and social factors^13^ ^,^ ^19^ ^,^ ^20^ ^,^ ^21^.

Most studies assessing the association between socioeconomic context and health outcomes primarily use the Human Development Index (HDI) and the Gini Index^19^ ^,^ ^22^. Although widely employed, these measures rely on aggregated indicators for large geographic areas, such as countries, states, and municipalities. This has been identified as a limitation, primarily because of the internal heterogeneity observed at these levels of aggregation^23^.

In 2019, researchers from the Centre for Data and Knowledge Integration for Health (Fiocruz Bahia) and the University of Glasgow (Scotland) developed the Brazilian Deprivation Index (BDI). Its objective is to characterize socioeconomic context in small areas based on indicators of income, education, and household conditions among the local population. The BDI provides information on socioeconomic status across Brazil at the census tract level, enabling the analysis of social inequalities in health^24^.

Given the aging of the population, understanding the role of social determinants of health among older adults has become increasingly important. As people age, the likelihood of physical, mental, and cognitive vulnerabilities increases, as does the onset or worsening of chronic and degenerative diseases^25^ ^,^ ^26^, potentially intensifying existing health inequalities.

Thus, considering the context of population aging in Brazil, characterized by the “feminization of aging” (a consequence of women’s longer life expectancy^27^) and by social inequalities regarding access to and use of health services, this study builds on previous literature by utilizing the BDI. Measured at the census tract level, this index overcomes the limitations associated with indicators aggregated across larger geographic areas. Accordingly, the objective of this study is to evaluate the contribution of socioeconomic context to health service utilization indicators among women aged 50 years and older, stratified by source of care (the Brazilian public health system, called Unified Health System or SUS versus private health insurance). Stratification by source of care is necessary because of differences in healthcare system organization, as this variable may act as an important confounder in the study. The study hypothesizes that, beyond individual-level factors, the local socioeconomic context plays a crucial role in understanding health service utilization among Brazilian women, whether they rely on SUS or have private health insurance. The findings aim to support the implementation and monitoring of public policies that ensure comprehensive, longitudinal care for women, enabling them to age with greater health and dignity.

## METHODS

### Study design and population

A cross-sectional observational study was conducted using baseline data from the Brazilian Longitudinal Study of Aging (ELSI-Brasil), collected between 2015 and 2016 from a nationally representative sample of the population aged 50 years or older. This manuscript was prepared in accordance with the Strengthening the Reporting of Observational Studies in Epidemiology (STROBE) guidelines for observational studies.

The ELSI-Brasil baseline sample included 9,412 residents from 70 municipalities across Brazil’s five macro-regions. To ensure representativeness of urban and rural areas in small, medium-sized, and large municipalities, ELSI-Brasil used a complex sampling design, combining the stratification of primary sampling units (municipalities), census tracts, and households. Further details are available in another publication^28^ and on the study website (https://elsi.cpqrr.fiocruz.br/). This study is part of the project “The influence of the physical and social environment on the health of older Brazilian adults: a longitudinal, multimethod, population-based study (ELSI-Urbe)”. The BDI^24^ ^,^ ^29^ was incorporated into the ELSI-Brasil database. Female participants from ELSI-Brasil were included in this study.

ELSI-Brasil was funded by the Ministry of Health: Department of Science and Technology of the Secretariat of Science, Technology, and Strategic Supplies (Grant Nos. 404965/2012-1 and TED 28/2017) and the Coordination of Older Adult Health in Primary Care, Department of Life Cycles of the Secretariat of Primary Health Care (Grant Nos. 20836, 22566, 23700, 25560, 25552, and 27510). ELSI-Urbe is funded by the Minas Gerais State Research Support Foundation (Project APQ-02425-21).

The study is coordinated by the Oswaldo Cruz Foundation (Minas Gerais) and the Federal University of Minas Gerais. It was approved by the Research Ethics Committee of the René Rachou Institute (Oswaldo Cruz Foundation), and the project is registered with Plataforma Brasil (Protocol No. 886.754). All participants who agreed to take part in the study provided informed consent by signing an informed consent form.

### Study variables

#### Indicators of health service utilization

The outcomes of interest were indicators of health service utilization, assessed based on mammography and Pap smear testing, cholesterol and blood glucose testing, and blood pressure measurement.

The analysis of mammography screening was based on Ministry of Health recommendations^30^ and included women aged 50 to 69 who answered the question: “When was the last time you had a mammogram or breast X-ray?” Responses were dichotomized into “two years or less” or “more than two years/never”.

For Pap smear screening, women aged 50 to 64 years were included, in accordance with Ministry of Health guidelines^30^. Participants were asked: “When was the last time you had a preventive screening test for cervical cancer (Pap smear)?” Responses were dichotomized into “3 years or less” or “more than 3 years/never had the test”.

The analyses of cholesterol and blood glucose testing included all women aged 50 years or older, as there is no consensus in the literature regarding the recommended frequency of these tests. These outcomes were assessed using the following questions, respectively: “When was the last time you had a blood test to measure your cholesterol?” and “When was the last time you had a test to measure your blood glucose (sugar)?” Responses were dichotomized into “3 years or less” or “more than 3 years/never had the test”.

Finally, in accordance with Ministry of Health guidelines^31^, blood pressure measurement was also assessed among all women aged 50 years or older. This outcome was measured using the question: “When was the last time your blood pressure was measured?” Responses were dichotomized into “within the last year” or “more than 1 year ago/never measured”.

Figure 1 presents a description of the sample included in the analysis for each test. Women who met the specific age criteria and had valid data for each outcome were considered eligible for the respective analyses.

**Figure 1.**
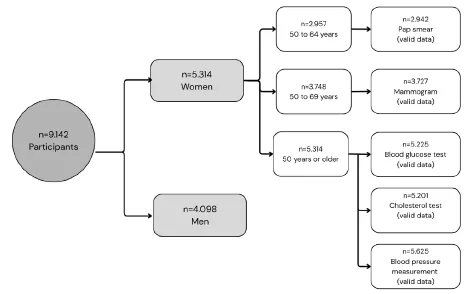
Sample selection flowchart and eligibility criteria (ELSI-Brasil, 2015-2016).

Source: Prepared by the authors on the basis of ELSI-Brasil data, 2015-2016.

#### Contextual socioeconomic indicator

Socioeconomic status was measured at the contextual level using the BDI. This index was developed based on data from the 2010 Census, and material deprivation estimates were generated for each Brazilian census tract based on three indicators: the percentage of households with a per capita income of up to half the minimum wage; the percentage of illiterate individuals aged seven years or older; and the percentage of households with inadequate access to basic sanitation and lacking garbage collection, piped water, a flush toilet, and a bathroom. In this study, the BDI was analyzed as a continuous variable, ranging from -3.12 to 15.59. Higher index values indicate greater deprivation^24^.

#### Other variables

Sample characterization was based on the following variables: self-reported race/skin color (white or other [Black, Brown, Asian, and Indigenous]), age group (50-59, 60-69, 70-79, or 80 years and older), educational attainment (<4 years, 4-7 years, or ≥8 years of schooling), household arrangement (living alone or not living alone), per capita household income (in tertiles: up to R$ 587.00, R$ 587.01 to R$ 1,050.00, and above R$ 1,050.00), and number of chronic diseases (none, one chronic disease, or ≥2 chronic diseases). For the latter, the study considered self-reported medical diagnoses of hypertension, diabetes, heart disease (heart attack, angina, or heart failure), stroke, chronic obstructive pulmonary disease, arthritis or rheumatism, depression, cancer, and chronic kidney failure.

The source of healthcare was assessed using the question: “Are you covered by a private health insurance plan from a company or a public agency?”. Responses were dichotomized as “yes” or “no”. This classification is based on the structure of the Brazilian health system, which comprises a public sector (SUS) and a supplementary private health insurance sector, each with distinct financing and access arrangements^32^.

### Data analysis

Sample characterization was based on frequency distributions with corresponding 95% confidence intervals. In unadjusted analyses, Pearson’s chi-square test with Rao-Scott correction was used to assess the statistical significance of the association between the independent variable and outcomes, stratified by healthcare source and accounting for the complex sampling design.

The association between the contextual-level variable (BDI) and the outcomes was analyzed using crude and adjusted multilevel logistic regression models, following evidence of significant variability in outcomes across census tracts. This variability was assessed via the intraclass correlation coefficient (ICC) and median odds ratio (MOR), which are standard measures for evaluating the relevance of geographic units to health outcomes^33^ ^,^ ^34^.

Potential confounders considered in this study included age group, household arrangement, self-reported race/skin color, and the number of self-reported chronic diseases. All analyses were stratified by healthcare source. To illustrate the association between the BDI and healthcare service utilization indicators, predicted probabilities of the outcomes across BDI levels were calculated and presented graphically.

Given that some census tracts (the level-2 units in the multilevel models) contained few women, particularly when considering the specific target populations for each outcome, a sensitivity analysis was conducted to assess the robustness of the findings. Multilevel models analyzing the association between the BDI and outcomes were refitted, including only census tracts with at least three eligible women. The same adjustment variables described above were included in these models.

All analyses were performed using Stata^®^ version 16.1, with a 5% significance level and accounting for sampling weights.

## Data availability statement:

The entire dataset supporting the results of this study is available from the corresponding author upon request. The dataset is not publicly available because of the integration of databases performed by the authors.

## RESULTS

The total sample consisted of 5,314 women aged 50 years or older, of whom 54.2% were aged 60 years or older. Regarding self-reported race/skin color, 57.3% identified as Black, Brown, Asian, or Indigenous. Approximately one in ten participants lived alone, and 34.5% had fewer than four years of schooling. Twenty-eight percent of the women reported a diagnosis of at least two chronic diseases. Regarding health service utilization, 74.2% of the women had undergone mammography within the previous two years, and 77.7% had undergone Pap smear testing within the previous three years. Blood glucose and cholesterol tests had been performed within the previous three years by 88.7 and 89.7% of participants, respectively. Blood pressure had been measured within the previous year for 94.0% of the women (Table 1).

**Table 1.** Characterization of the sample, total and stratified by private health insurance coverage among older women. (ELSI-Brasil, 2015-2016).

Variables	Total sample % (95%CI)	With health insurance % (95%CI)	Without health insurance % (95%CI)	p-value*
Sociodemographic characterization				
Age group (years)				
50-59	45.8 (42.6-49.0)	39.6 (35.4-44.0)	47.9 (44.6-51.4)	<0.001
60-69	29.6 (27.9-31.4)	29.2 (26.0-32.5)	29.8 (27.9-31.8)	
70-79	16.4 (14.8-18.1)	19.4 (17.0-22.0)	15.4 (13.7-17.2)	
80 and older	8.2 (6.8-9.6)	11.8 (9.6-14.6)	6.8 (5.6-8.3)	
Self-reported skin color				
White	42.7 (36.6-48.9)	54.7 (49.2-60.1)	38.5 (31.9-45.4)	<0.001
Other	57.3 (51.1-63.3)	45.3 (39.9-50.8)	61.5 (54.6-68.0)	
Living alone				
Yes	10.7 (9.6-12.0)	12.9 (10.9-15.3)	9.9 (8.9-11.1)	<0.001
No	89.3 (88.0-90.4)	87.1 (84.7-89.1)	90.0 (88.7-91.0)	
Education (years of study)				
<4	34.5 (31.1-38.0)	17.6 (14.4-21.2)	40.2 (36.4-44.3)	<0.001
4-7	31.2 (28.6-34.0)	25.8 (23.0-28.9)	33.1 (29.8-36.6)	
8 and more	34.3 (31.4-37.3)	56.6 (51.8-61.2)	26.6 (23.8-29.6)	
Per capital household income				
Tertile 1	35.5 (31.6-39.7)	21.3 (18.2-24.7)	40.4 (35.8-45.2)	<0.001
Tertile 2	33.5 (31.6-35.4)	24.2 (21.3-27.4)	36.7 (34.3-39.1)	
Tertile 3	31.0 (27.8-34.4)	54.5 (50.1-58.9)	22.9 (19.9-26.2)	
Health condition				
Number of chronic diseases^†^				
No chronic diseases	39.1 (36.6-41.6)	36.8 (33.4-40.3)	39.8(36.9-42.8)	0.354
1 chronic disease	33.1 (31.9-34.4)	34.8(31.7-38.1)	32.6(31.1-34.1)	
2 or more chronic diseases	27.8 (25.6-30.1)	28.4(25.2-31.9)	27.6(24.9-30.5)	
Use of health services				
Pap smear (last 3 years)^‡^	77.7 (75.6-79.6)	86.3 (82.9-89.1)	75.1 (72.5-77.5)	<0.001
Mammogram (last 2 years)^§^	74.2 (71.7-76.4)	80.9 (77.4-83.9)	71.7 (68.6-74.7)	<0.001
Blood glucose (last 3 years)	88.7 (87.0-90.2)	94.4 (92.4-95.9)	86.8 (84.7-88.6)	<0.001
Cholesterol (last 3 years)	89.7 (87.9-91.3)	95.4 (93.6-96.8)	87.8 (85.5-89.7)	<0.001
Blood pressure (within last year)	94.0 (93.0-94.8)	95.8 (94.4-96.9)	93.4 (92.1-94.5)	0.012

Source: Prepared by authors on the basis of ELSI-Brasil data.

CI: confidence interval.

*p-value for the Rao-Scott corrected χ^2^ test. ^†^Self-reported medical diagnosis of the following conditions: hypertension, diabetes, heart disease, stroke, chronic obstructive pulmonary disease, arthritis or rheumatism, depression, cancer, chronic renal failure; ^‡^50 to 64 years; ^§^50 to 69 years.

Private health insurance coverage was reported by 25.3% of the women. With the exception of the number of chronic diseases, all other variables were statistically associated with private health insurance coverage; health service utilization was higher among women with private insurance, as shown in Table 1.

The ICC and MOR are important measures for assessing the relevance of context to outcomes, indicating, respectively, the proportion of variability explained by the context and its effect on the outcome in terms of odds ratio. In this study, the ICC and MOR estimates demonstrated the relevance of the context (census tract of residence) to health service utilization among older women. For example, the ICC estimates showed that 22% of the variability in cholesterol testing within the previous three years could be explained by the census tract. Furthermore, the MOR estimates indicated up to 2.54-fold increase in the odds of a participant undergoing cholesterol testing within the previous three years if she resided in a census tract with lower deprivation (Table 2).

**Table 2.** Intraclass correlation coefficient and median odds ratio estimated after null model adjustment among older women (ELSI-Brasil, 2015-2016).

Variables Sociodemographic	Pap smear* (last 3 years)	Mammogram^†^ (last 2 years)	Blood glucose (last 3 years)	Cholesterol (last 3 years)	Blood pressure (within last year)
General					
ICC	0.15	0.12	0.18	0.22	0.19
	(0.11-0.21)	(0.08-0.18)	(0.14-0.24)	(0.17-0.29)	(0.13-0.28)
Median OR	2.07	1.91	2.28	2.54	2.33
	(1.76-2.38)	(1.64-2.19)	(1.99-2.57)	(2.14-2.93)	(1.84-2.81)
Without health insurance					
ICC	0.17	0.14	0.16	0.21	0.24
	(0.12-0.24)	(0.09-0.21)	(0.12-0.22)	(0.15-0.28)	(0.16-0.35)
Median OR	2.22	2.02	2.14	2.41	2.65
	(1.86-2.56)	(1.69-2.35)	(1.84-2.44)	(2.00-2.82)	(1.99-3.31)
Enrollment in a private health plan					
ICC	0.38	0.32	0.43	0.43	0.40
	(0.21-0.58)	(0.20-0.47)	(0.28-0.60)	(0.24-0.64)	(0.21-0.63)
Median OR	3.83	3.27	4.56	4.46	4.15
	(1.72-5.93)	(2.02-4.52)	(2.27-6.85)	(1.63-7.28)	(1.36-6.93)

Source: Prepared by authors on the basis of ELSI-Brasil data.

ICC: intraclass correlation coefficient; MOR: median odds ratio.

*50 to 64 years; ^†^50 to 69 years.

Table 3 presents the results of the crude and adjusted analyses of the associations between the BDI and the outcomes, stratified by private health insurance coverage.

**Table 3.** Association (odds ratio [95% confidence interval]) between the Brazilian Deprivation Index and healthcare service use among older women (ELSI-Brasil, 2015-2016).

Model	Pap smear* (last 3 years)	Mammogram^†^ (last 2 years)	Blood glucose (last 3 years)	Cholesterol (last 3 years)	Blood pressure (within last year)
With health insurance					
Not adjusted	0.85	0.99	**0.79**	**0.73**	0.84
	(0.73-1.00)	(0.86-1.13)	**(0.68-0.92)**	**(0.63-0.86)**	(0.68-1.04)
Adjusted^‡^	0.87	1.02	**0.82**	**0.71**	0.89
	(0.73-1.03)	(0.87-1.21)	**(0.69-0.96)**	**(0.59-0.87)**	(0.70-1.12)
Without health insurance					
Not adjusted	0.97	0.99	**0.89**	**0.88**	1.03
	(0.91-1.02)	(0.94-1.04)	**(0.86-0.93)**	**(0.84-0.93)**	(0.96-1.09)
Adjusted^‡^	0.97	0.98	**0.91**	**0.89**	1.05
	(0.91-1.03)	(0.92-1.03)	**(0.87-0.96)**	**(0.85-0.95)**	(0.98-1.13)

Source: Prepared by authors on the basis of ELSI-Brasil data.

*50 to 64 years; ^†^50 to 69 years; ^‡^Estimates adjusted by age group, self-reported skin color, living alone, and number of chronic diseases (self-reported medical diagnosis of the following conditions: hypertension, diabetes, heart disease, stroke, chronic obstructive pulmonary disease, arthritis or rheumatism, depression, cancer, chronic renal failure).

Values in bold represent statistically significant associations (p<0.05).

In the adjusted analyses, significant associations were observed among women with and without private health insurance, respectively, between the BDI and blood glucose testing (odds ratio [OR] 0.82; 95% confidence interval [95%CI] 0.69-0.96 and OR 0.91; 95%CI 0.87-0.96) and cholesterol testing (OR 0.71; 95%CI 0.59-0.87 and OR 0.89; 95%CI 0.85-0.95) within the previous three years. Women residing in census tracts with lower deprivation had higher odds of undergoing these tests.

The adjustments did not substantially alter the findings. There was no significant association between the BDI and Pap smear testing, mammography, or blood pressure measurement, regardless of the source of care. Figure 2 illustrates these results using predicted probabilities of undergoing the tests according to BDI and private health insurance coverage, adjusted for age group, self-reported race/skin color, household arrangement, and number of chronic diseases.

**Figure 2.**
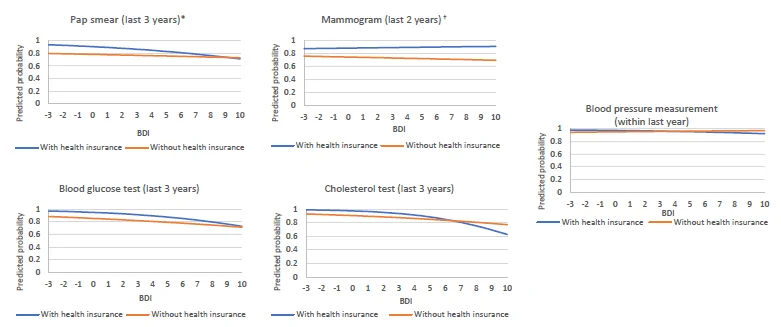
Predicted probability^‡^ of undergoing preventive health screenings according to the Brazilian Deprivation Index and source of healthcare, among older women (ELSI-Brasil, 2015-2016).

BDI: Brazilian Deprivation Index.

*50 to 64 years; ^†^50 to 69 years; ^‡^Adjusted for age group, self-reported skin color, living alone, and number chronic diseases.

Source: Prepared by the authors on the basis of ELSI-Brasil data.

## DISCUSSION

This study investigated inequities in health service utilization among women aged 50 years or older, specifically regarding mammography, Pap smear testing, cholesterol and blood glucose testing, and blood pressure measurement. The main findings showed that women living in census tracts with greater socioeconomic deprivation were less likely to have undergone blood glucose and cholesterol testing, regardless of whether they had private health insurance.

It is well established that individuals with private health insurance have more medical consultations, experience shorter waiting times for appointments, and have greater access to specialist care^35^. Brazilian studies have shown that women with private health insurance are more likely to undergo mammography and Pap smear testing^10^ ^,^ ^13^. A similar pattern has been observed in the United States, where lack of health insurance was associated with 31 and 41% lower rates of mammography and Pap smear testing, respectively, as well as a 24% lower rate of cholesterol testing^36^. In Colombia, a lower likelihood of undergoing mammography and blood glucose testing has also been observed among individuals without health insurance. Conversely, in Mexico, having health insurance facilitates cholesterol and blood glucose testing as well as blood pressure monitoring^37^ ^,^ ^38^.

Beyond individual-level factors, several studies have demonstrated that socioeconomic context, commonly assessed using indices such as the HDI and Gini coefficient, is also a determinant of health service utilization^19^ ^,^ ^22^.

In this study, the socioeconomic context was characterized using the BDI, measured at the census tract level^29^, which distinguishes it from other indices. Analyses of the association between the BDI and healthcare service utilization indicators among older Brazilian women showed a higher likelihood of undergoing cholesterol and blood glucose testing among those living in areas with lower levels of deprivation, regardless of individual characteristics or source of care. Studies using contextual socioeconomic measures at the census tract level to investigate health inequalities remain scarce in the literature. In contrast, studies evaluating the association of the HDI and Gini coefficient with mammography and Pap smear uptake have shown that higher HDI values in state capitals were positively associated with higher screening rates. Furthermore, greater income inequality, as measured by the Gini coefficient, and lower HDI were associated with access barriers and lower occurrence of these screenings^13^ ^,^ ^21^. International evidence reinforces the association between socioeconomic context and women’s healthcare utilization. In Texas, women living in poorer municipalities underwent fewer preventive Pap smear tests^39^. Similarly, in Ontario, Canada, residents of neighborhoods in the lowest income quintile underwent fewer mammograms and cholesterol and blood glucose tests^40^.

In the present study, no significant associations were observed between the BDI and undergoing Pap smear testing or mammography or having blood pressure measured. This suggests that the uptake of these procedures is not determined by the level of deprivation in the census tract, in contrast to the findings observed for blood glucose and cholesterol testing. Despite ongoing debate in the literature regarding the effectiveness of health campaigns, particularly with respect to their reach among the target population^41^ ^,^ ^42^ ^,^ ^43^, one possible explanation for these findings is the effectiveness of campaigns aimed at raising awareness of the importance of mammography and Pap smear testing. These campaigns are well-established and widely disseminated, potentially mitigating the influence of contextual socioeconomic factors on screening uptake. Blood pressure measurement, in turn, is a common procedure across the population; it can be performed during any medical consultation^44^ and is available at various healthcare facilities and other settings, such as private pharmacies that offer this service, as well as through the purchase of home blood pressure monitors. In contrast, similar strategies are not available for blood glucose and cholesterol testing, making access to these tests more dependent on contextual socioeconomic factors, which may act as either facilitators or barriers to healthcare utilization.

Using the BDI to characterize the contextual socioeconomic status of the census tract of residence made it possible to assess the association between women’s proximal socioeconomic environment and their use of health services. Unlike contextual analyses based on larger geographic areas^19^ ^,^ ^23^ ^,^ ^45^, such as municipalities or states, which entail greater internal heterogeneity, the evidence presented here can help health managers identify areas where women are less likely to undergo these tests, thereby facilitating the implementation of more targeted actions and strategies.

This study contributes to the existing literature. It was conducted using a nationally representative sample of the non-institutionalized Brazilian population aged 50 years or older, residing in both urban and rural areas across municipalities of different sizes (small, medium, and large). The data were analyzed using an appropriate statistical methodology, specifically multilevel modeling, and an index developed to assess the contextual socioeconomic status of the census tract.

However, an important limitation is the potential for information bias, as the outcomes were self-reported. Individuals affected by a health condition or disease tend to recall undergoing these tests more readily than those who are not affected. Furthermore, because of the cross-sectional design, a causal relationship between the variables cannot be established. It is also worth noting that the BDI was calculated using 2010 Census data, which may not reflect the current socioeconomic status of the Brazilian population.

In summary, the results revealed lower rates of blood glucose and cholesterol testing among older Brazilian women living in areas of greater socioeconomic deprivation. These findings highlight the need for public policies and interventions targeting older Brazilian women who rely on the public health system for care and reside in highly deprived census tracts; such measures are essential to reducing inequities in health service utilization. Moreover, the findings underscore the importance of broadening the scope of women’s health policies beyond gynecological cancer screening to include the routine assessment of tests such as blood glucose and cholesterol, thereby ensuring comprehensive care that also addresses the social determinants of health.
