# Supplementary material for: Association between socioeconomic context and getting preventive screenings among Brazilian women aged 50 years and older
Source: Rev Bras Epidemiol. 2026 Sep 28;29:e260051. doi: 10.1590/1980-549720260051 (PMC13619126; doi:10.1590/1980-549720260051)
Supplement: Translation

# Translation: Associação entre contexto socioeconômico e realização de exames preventivos entre mulheres brasileiras com 50 anos ou mais

**DOI:** 10.1590/1980-549720260051.2

**Authors:** Poliana Fialho de Carvalho, Luciana de Souza Braga, Sérgio Viana Peixoto, Waleska Teixeira Caiaffa, Maria Fernanda de Lima-Costa, Juliana Vaz de Melo Mambrini

**Affiliations:** I Instituto René Rachou - Fiocruz Minas, Belo Horizonte, Minas Gerais, Brasil.; II Universidade Federal de Minas Gerais, Belo Horizonte, Minas Gerais, Brasil.

**Keywords:** Uso de serviços de saúde, Saúde da mulher, Mensuração das desigualdades em saúde, Estudos transversais

## Abstract

**Objetivo:**

Avaliar a associação entre o contexto socioeconômico e indicadores de uso dos serviços de saúde entre mulheres com 50 anos ou mais, segundo a fonte de atenção (usuárias exclusivas do Sistema Único de Saúde [SUS] versus de plano de saúde).

**Métodos:**

Estudo transversal com dados da linha de base do Estudo Longitudinal da Saúde dos Idosos Brasileiros (2015-2016), com amostra representativa da população com 50 anos ou mais. Foram analisados os seguintes indicadores de uso dos serviços de saúde: realização de mamografia, Papanicolau, dosagem de colesterol e glicemia e aferição da pressão arterial. A condição socioeconômica contextual foi medida pelo Índice Brasileiro de Privação (IBP). Utilizou-se modelo logístico multinível, bruto e ajustado, depois de constatar variabilidade significativa entre setores censitários.

**Resultados:**

A amostra incluiu 5.314 mulheres, sendo 54,2% com 60 anos ou mais. Observou-se um gradiente socioeconômico na realização de exames de glicemia e colesterol, com menor probabilidade de realização entre mulheres de áreas de maior privação. Esse padrão persistiu independentemente da fonte de atenção, sendo identificado tanto entre usuárias exclusivas do SUS quanto entre beneficiárias de plano privado (glicemia: odds ratio - OR 0,82, intervalo de confiança de 95% - IC95% 0,69-0,96 e OR 0,91, IC95% 0,87-0,96; colesterol: OR 0,71, IC95% 0,59-0,87 e OR 0,89, IC95% 0,85-0,95, respectivamente).

**Conclusão:**

Os resultados revelam iniquidades nas dosagens de glicemia e colesterol por mulheres com 50 anos ou mais, especialmente entre aquelas que dependem do SUS e vivem em áreas de pior contexto socioeconômico. Reforça-se a necessidade de políticas públicas focadas nas mulheres para reduzir as iniquidades presentes na utilização dos serviços de saúde.

## INTRODUÇÃO

Apesar dos avanços recentes, a desigualdade social em saúde persiste no Brasil^1^. Estudos mostram que essas desigualdades também se manifestam na realização de exames preventivos. Aqueles com maior renda, alta escolaridade, residentes em áreas urbanas e nas regiões mais desenvolvidas, além daqueles afiliados a planos privados de saúde, são os que mais utilizam esses serviços^2^ ^,^ ^3^ ^,^ ^4^.

Tais desigualdades repercutem na ocorrência e no manejo das doenças crônicas não transmissíveis (DCNT), um importante problema de saúde pública^5^ ^,^ ^6^ ^,^ ^7^. Elas possuem maior incidência e são a maior causa de morbimortalidade globalmente, responsáveis por 38 milhões de mortes anuais, 75% delas em países de baixa e média renda^7^ ^,^ ^8^. No Brasil, 54,7% dos óbitos ocorridos em 2019 foram por DCNT^5^. Entre as mulheres, as duas principais causas de óbito foram doenças do aparelho circulatório e neoplasias^9^.

Dentre as neoplasias, o câncer de mama é o que mais mata as mulheres, e o câncer de colo de útero ocupa a terceira posição. Mulheres casadas, com alta escolaridade e plano privado de saúde, possuem maior probabilidade de terem realizado a mamografia e Papanicolau, exames para diagnóstico desses cânceres^10^ ^,^ ^11^ ^,^ ^12^ ^,^ ^13^.

No Brasil, a criação da Estratégia Saúde da Família (ESF) contribuiu positivamente para diversos indicadores de saúde, incluindo os relacionados às DCNT, como a redução de internações por condições sensíveis à Atenção Primária e da mortalidade cardiovascular, tornando-a uma importante política pública para mitigar as consequências das desigualdades em saúde^14^ ^,^ ^15^ ^,^ ^16^. Contudo, o país ainda é marcado pelas desigualdades sociais no acesso e no uso de serviços de saúde^17^ ^,^ ^18^.

Além dos fatores socioeconômicos individuais, estudos apontam um papel importante e independente do contexto socioeconômico, salientando que os diferenciais em saúde também são determinados por fatores macroeconômicos e sociais^13^ ^,^ ^19^ ^,^ ^20^ ^,^ ^21^.

A maioria dos estudos que avalia a associação entre contexto socioeconômico e desfechos de saúde utiliza principalmente o Índice de Desenvolvimento Humano (IDH) e o Índice de Gini^19^ ^,^ ^22^. Apesar de amplamente empregados, eles fazem uso de indicadores agregados para áreas geográficas muito extensas, como países, estados e municípios. Isso tem sido apontado como uma limitação, devida principalmente à heterogeneidade interna observada nesses níveis de agregação^23^.

Em 2019, pesquisadores do Centro de Integração de Dados e Conhecimentos para Saúde (Fiocruz Bahia) e da Universidade de Glasgow-Escócia desenvolveram o Índice Brasileiro de Privação (IBP), cujo objetivo é caracterizar o contexto socioeconômico em áreas pequenas, baseando-se em indicadores de renda, escolaridade e condições domiciliares da população do território. O IBP informa a posição socioeconômica em todo o Brasil em nível de setores censitários, viabilizando a análise das desigualdades sociais em saúde^24^.

Diante do envelhecimento populacional, torna-se mais relevante entender o papel dos determinantes sociais da saúde em adultos mais velhos. À medida que se envelhece, aumenta-se a chance de ocorrência de vulnerabilidades físicas, mentais e cognitivas, além do surgimento ou agravamento de doenças crônicas e degenerativas^25^ ^,^ ^26^, podendo-se intensificar as desigualdades em saúde já existentes.

Dessa forma, considerando-se o cenário do envelhecimento populacional brasileiro, marcado pela feminização da velhice, consequência da maior expectativa de vida de mulheres^27^, e pela desigualdade social no acesso e uso dos serviços de saúde, este estudo avança em relação à literatura prévia ao utilizar o IBP. A adoção desse índice, mensurado em nível de setor censitário, permite superar as limitações de indicadores agregados em áreas geográficas de maior extensão territorial. Assim, o objetivo deste trabalho é avaliar a contribuição do contexto socioeconômico para os indicadores de uso dos serviços de saúde entre mulheres com 50 anos e mais, segundo a fonte de atenção (usuários exclusivos do SUS ou afiliação a plano privado de saúde). A estratificação pela fonte de atenção é necessária em razão das diferenças de organização dos sistemas, podendo ser um importante fator de confusão para o objeto de estudo. A hipótese deste trabalho é que, para além dos fatores de nível individual, o contexto socioeconômico local exerce um papel importante para a compreensão acerca do uso dos serviços de saúde pelas mulheres brasileiras, tanto para as usuárias do SUS quanto para aquelas que possuem plano de saúde. Os resultados visam auxiliar a implementação e o monitoramento de políticas públicas que garantam um cuidado longitudinal integral às mulheres, permitindo-lhes um envelhecer com mais saúde e dignidade.

## MÉTODOS

### Delineamento do estudo e população

Trata-se de um estudo observacional transversal, que utiliza dados da linha de base do Estudo Longitudinal da Saúde dos Idosos Brasileiros (ELSI-Brasil), coletados entre 2015 e 2016, conduzido em amostra representativa da população com 50 anos ou mais. Este manuscrito foi elaborado de acordo com as recomendações do Strengthening the reporting of observational studies in epidemiology (STROBE) para estudos observacionais.

Participaram da amostra da linha de base do ELSI-Brasil 9.412 residentes em 70 municípios das cinco macrorregiões brasileiras. Para garantir a representatividade das áreas urbanas e rurais dos municípios de pequeno, médio e grande porte, o ELSI-Brasil utilizou delineamento de amostra complexa, combinando a estratificação de unidades primárias de amostragem (municípios), setores censitários e domicílios. Maiores detalhes podem ser vistos em outra publicação^28^ e no site da pesquisa (https://elsi.cpqrr.fiocruz.br/). Este trabalho integra o projeto “A influência do ambiente físico e social na saúde de adultos brasileiros mais velhos: estudo de base populacional longitudinal multimétodos (ELSI-Urbe)”. Ao banco de dados do ELSI-Brasil foi acrescentado o IBP^24^ ^,^ ^29^. Neste estudo foram incluídos os participantes do ELSI-Brasil do sexo feminino.

O ELSI-Brasil foi financiado pelo Ministério da Saúde: Departamento de Ciência e Tecnologia da Secretaria de Ciência, Tecnologia e Insumos Estratégicos (Processos: 404965/2012-1 e TED 28/2017) - Coordenação de Saúde da Pessoa Idosa na Atenção Primária, Departamento dos Ciclos da Vida da Secretaria de Atenção Primária à Saúde (Processos: 20836, 22566, 23700, 25560, 25552 e 27510). O ELSI-Urbe é financiado pela Fundação de Amparo à Pesquisa do Estado de Minas Gerais (Projeto APQ-02425-21).

O estudo é coordenado pela Fundação Oswaldo Cruz/Minas Gerais e pela Universidade Federal de Minas Gerais. Foi aprovado pelo Comitê de Ética em Pesquisa do Instituto René Rachou da Fundação Oswaldo Cruz e o processo está cadastrado na Plataforma Brasil (Protocolo n^o^ 886.754). Todos os que concordaram em participar da pesquisa registraram seu consentimento por meio da assinatura do Termo de Consentimento Livre e Esclarecido.

### Variáveis do estudo

#### Indicadores de uso dos serviços de saúde

Os desfechos de interesse são os indicadores de uso dos serviços de saúde, avaliados por meio da realização dos exames de mamografia e Papanicolau, dosagem de colesterol e glicemia e aferição da pressão arterial.

A análise do exame de mamografia foi realizada com base na recomendação do Ministério da Saúde^30^ e considerou as mulheres de 50 a 69 anos que responderam à pergunta: “Quando foi a última vez que a Sra. fez uma mamografia ou raio X de mama?”, com respostas dicotomizadas em “dois anos ou menos” ou “mais de dois anos/nunca”.

Para o exame de Papanicolau, foram incluídas as participantes com idade entre 50 e 64 anos, conforme orientação do Ministério da Saúde^30^, que responderam à pergunta: “Quando foi a última vez que a Sra. fez um exame preventivo para câncer de colo do útero (exame do Papanicolau)?”. As respostas foram dicotomizadas em “3 anos ou menos” ou “mais de 3 anos/nunca realizou”.

Nas análises das dosagens de colesterol e glicemia, foram incluídas todas as mulheres com 50 anos ou mais, não existindo um consenso na literatura com relação à indicação de periodicidade para a realização desses exames. Esses desfechos foram avaliados, respectivamente, por meio das seguintes perguntas: “Qual foi a última vez que a Sra. fez exame de sangue para medir o colesterol?” e “Qual foi a última vez que a Sra fez exame para medir a glicemia (açúcar) no sangue?”, e as respostas foram dicotomizadas em “3 anos ou menos” ou “mais de 3 anos/nunca realizou”.

Por fim, seguindo as orientações do Ministério da Saúde^31^, a aferição da pressão arterial foi também avaliada para todas as mulheres com 50 anos ou mais. Esse desfecho foi mensurado pela pergunta: “Qual foi a última vez que a sua pressão arterial foi medida?”, com respostas dicotomizadas em “até 1 ano” ou “mais de 1 ano/nunca aferiu”.

A Figura 1 apresenta a descrição da amostra incluída na análise de cada exame. Foram selecionadas as mulheres do grupo etário específico e com informação válida para cada desfecho.

**Figura 1.** Fluxograma de seleção da amostra e critérios de elegibilidade (ELSI-Brasil, 2015-2016).

Fonte: Elaboração própria, com base em dados do ELSI-Brasil.

#### Indicador socioeconômico contextual

A condição socioeconômica foi mensurada, no nível contextual, utilizando o IBP. Esse índice foi desenvolvido com base em dados do Censo 2010, e estimativas de privação material foram produzidas para cada setor censitário brasileiro, com base em três indicadores: percentual de domicílios com renda per capita de até ½ salário-mínimo, percentual de indivíduos analfabetos com sete anos ou mais de idade e percentual de domicílios com acesso inadequado ao saneamento básico e sem coleta de lixo, água encanada, vaso sanitário e banheiro. Neste estudo, o IBP foi analisado em sua forma contínua, variando entre -3,12 e 15,59. Maiores valores do índice indicam maior privação^24^.

#### Outras variáveis

A caracterização da amostra foi realizada por meio das variáveis cor autorreferida (branca ou outra [preta, parda, amarela e indígena]), faixa etária (50-59, 60-69, 70-79 ou 80 anos e mais), escolaridade (<4 anos, 4-7 anos ou ≥8 anos de estudo), arranjo domiciliar (mora só ou não mora só), renda domiciliar per capita (em tercis: até R$ 587,00, R$ 587,01 a R$ 1.050,00 e superior a R$ 1.050,00) e número de doenças crônicas (nenhuma doença, uma doença crônica ou ≥2 doenças crônicas), sendo, para esta última, considerado o autorrelato de diagnóstico médico para hipertensão arterial, diabetes, doenças cardíacas (infarto, angina, insuficiência cardíaca), acidente vascular cerebral, doença pulmonar obstrutiva crônica, artrite ou reumatismo, depressão, câncer e insuficiência renal crônica.

A fonte de atenção à saúde foi avaliada por meio da pergunta: “A Sra. possui plano de saúde particular, de empresa ou órgão público?”, com respostas dicotomizadas em “sim” ou “não”. Essa classificação fundamenta-se na estrutura do sistema de saúde brasileiro, composto de um setor público (SUS) e um subsetor suplementar de planos privados de saúde, com dinâmicas distintas de financiamento e acesso^32^.

### Análise dos dados

A caracterização da amostra baseou-se em distribuições de frequência, com respectivos intervalos de 95% de confiança. Nas análises não ajustadas, foi utilizado o teste χ^2^ de Pearson, com correção de Rao-Scott, para examinar a significância estatística da associação entre as variável independente e os desfechos, de acordo com a fonte de atenção à saúde e considerando-se o efeito do desenho amostral.

A associação entre a variável de nível contextual (IBP) e os desfechos foi analisada pelo modelo logístico multinível, bruto e ajustado por potenciais fatores de confusão, após evidência de variabilidade significativa dos desfechos entre os setores censitários, por meio da avaliação do coeficiente de correlação intraclasse (ICC) e da mediana do odds ratio (MOR), medidas clássicas na avaliação da relevância das unidades geográficas para os desfechos de saúde^33^ ^,^ ^34^.

Neste estudo, foram considerados como potenciais fatores de confusão: faixa etária, arranjo domiciliar, cor autorreferida e número de doenças crônicas autorrelatadas. Todas as análises foram estratificadas segundo a fonte de atenção. Para ilustrar a associação entre o IBP e os indicadores de uso de serviços de saúde, foram calculadas probabilidades preditas dos desfechos segundo o IBP. Esses resultados foram apresentados graficamente.

Considerando-se que alguns setores censitários (unidade de nível 2 nos modelos multiníveis) possuem poucas mulheres na amostra, especialmente quando consideradas as especificidades de cada desfecho com relação ao público-alvo, foi realizada análise de sensibilidade com o objetivo de avaliar a robustez dos resultados apresentados. Modelos multiníveis para a análise da associação entre o IBP e os desfechos foram novamente ajustados, considerando-se apenas os setores censitários com pelo menos três mulheres elegíveis. Nessa análise, foram consideradas as mesmas variáveis de ajuste já citadas anteriormente.

Todas as análises foram realizadas no software Stata^®^ versão 16.1, considerando-se o nível de 5% de significância e o peso amostral.

## Declaração de disponibilidade de dados:

Todo o conjunto de dados que dá suporte aos resultados deste estudo está disponível mediante solicitação ao autor correspondente. O conjunto de dados não está publicamente disponível devido à integração de bancos de dados realizados pelos autores.

## RESULTADOS

A amostra total foi composta de 5.314 mulheres com 50 anos ou mais, sendo 54,2% com 60 anos ou mais. Quanto à cor autorreferida, 57,3% declararam-se pretas, pardas, amarelas ou indígenas. Aproximadamente uma em cada dez participantes morava só e 34,5% tinham menos de quatro anos de estudo. Vinte e oito por cento das mulheres reportaram diagnóstico de pelo menos duas doenças crônicas. Com relação ao uso de serviços de saúde, observou-se que 74,2% das mulheres realizaram o exame de mamografia nos últimos dois anos e 77,7% o de Papanicolau nos últimos três. Os exames de glicemia e colesterol foram realizados nos últimos três anos por 88,7 e 89,7% das participantes, respectivamente. A pressão arterial foi aferida há menos de um ano por 94,0% das mulheres (Tabela 1).

**Tabela 1.** Caracterização da amostra, total e estratificada pela afiliação a plano privado de saúde entre mulheres mais velhas (ELSI Brasil, 2015-2016).

Variáveis	Amostra Total % (IC95%)	Com plano de saúde % (IC95%)	Sem plano de saúde % (IC95%)	Valor p*
Caracterização sociodemográfica				
Faixa etária (anos)				
50-59	45,8 (42,6-49,0)	39,6 (35,4-44,0)	47,9 (44,6-51,4)	<0,001
60-69	29,6 (27,9-31,4)	29,2 (26,0-32,5)	29,8 (27,9-31,8)	
70-79	16,4 (14,8-18,1)	19,4 (17,0-22,0)	15,4 (13,7-17,2)	
80 e mais	8,2 (6,8-9,6)	11,8 (9,6-14,6)	6,8 (5,6-8,3)	
Cor autorreferida				
Branca	42,7 (36,6-48,9)	54,7 (49,2-60,1)	38,5 (31,9-45,4)	<0,001
Outra	57,3 (51,1-63,3)	45,3 (39,9-50,8)	61,5 (54,6-68,0)	
Mora só				
Sim	10,7 (9,6-12,0)	12,9 (10,9-15,3)	9,9 (8,9-11,1)	<0,001
Não	89,3 (88,0-90,4)	87,1 (84,7-89,1)	90,0 (88,7-91,0)	
Escolaridade (anos de estudo)				
<4	34,5 (31,1-38,0)	17,6 (14,4-21,2)	40,2 (36,4-44,3)	<0,001
4-7	31,2 (28,6-34,0)	25,8 (23,0-28,9)	33,1 (29,8-36,6)	
8 e mais	34,3 (31,4-37,3)	56,6 (51,8-61,2)	26,6 (23,8-29,6)	
Renda domiciliar per capita				
Tercil 1	35,5 (31,6-39,7)	21,3 (18,2-24,7)	40,4 (35,8-45,2)	<0,001
Tercil 2	33,5 (31,6-35,4)	24,2 (21,3-27,4)	36,7 (34,3-39,1)	
Tercil 3	31,0 (27,8-34,4)	54,5 (50,1-58,9)	22,9 (19,9-26,2)	
Condição de saúde				
Número de doenças crônicas^†^				
Nenhuma doença crônica	39,1 (36,6-41,6)	36,8 (33,4-40,3)	39,8(36,9-42,8)	0,354
1 doença crônica	33,1 (31,9-34,4)	34,8(31,7-38,1)	32,6(31,1-34,1)	
2 ou mais doenças crônicas	27,8 (25,6-30,1)	28,4(25,2-31,9)	27,6(24,9-30,5)	
Uso dos serviços de saúde				
Papanicolau (últimos 3 anos)^‡^	77,7 (75,6-79,6)	86,3 (82,9-89,1)	75,1 (72,5-77,5)	<0,001
Mamografia (últimos 2 anos)^§^	74,2 (71,7-76,4)	80,9 (77,4-83,9)	71,7 (68,6-74,7)	<0,001
Glicemia (últimos 3 anos)	88,7 (87,0-90,2)	94,4 (92,4-95,9)	86,8 (84,7-88,6)	<0,001
Colesterol (últimos 3 anos)	89,7 (87,9-91,3)	95,4 (93,6-96,8)	87,8 (85,5-89,7)	<0,001
Pressão Arterial (até 1 ano)	94,0 (93,0-94,8)	95,8 (94,4-96,9)	93,4 (92,1-94,5)	0,012

Fonte: Elaboração própria, com base em dados do ELSI-Brasil.

IC: intervalo de confiança.

*Valor p do teste χ^2^ com correção de Rao-Scott; ^†^Autorrelato de diagnóstico médico para as seguintes doenças: hipertensão arterial, diabetes, doença cardíaca, acidente vascular cerebral, doença pulmonar obstrutiva crônica, artrite ou reumatismo, depressão, câncer, insuficiência renal crônica; ^‡^50 a 64 anos; ^§^50 a 69 anos.

A afiliação a plano privado de saúde foi declarada por 25,3% das mulheres. Com exceção do número de doenças crônicas, todas as outras variáveis foram estatisticamente associadas com a afiliação a plano privado de saúde, sendo maior a utilização dos serviços de saúde entre as mulheres com plano de saúde, conforme pode ser visto na Tabela 1.

O coeficiente de correlação intraclasse (ICC) e a mediana do odds ratio (MOR) são medidas importantes para avaliação da relevância do contexto para os desfechos, mostrando, respectivamente, a proporção da variabilidade explicada pelo contexto e seu efeito no desfecho em termos de odds ratio. Neste estudo, as estimativas do ICC e da MOR evidenciaram a significância do contexto (setor censitário de residência) para o uso de serviços de saúde por mulheres mais velhas. As estimativas do ICC mostraram, por exemplo, que 22% da variabilidade de realização do exame de colesterol nos últimos três anos pode ser explicada pelo setor censitário. Além disso, estimativas da MOR apontaram aumento de até 2,54 na chance de uma participante realizar a dosagem de colesterol nos últimos três anos caso ela residisse em setor censitário de menor privação (Tabela 2).

**Tabela 2.** Coeficiente de correlação intraclasse e mediana da odds ratio estimados após ajuste de modelo nulo entre mulheres mais velhas (ELSI Brasil, 2015-2016).

Variáveis Sociodemográficas	Papanicolau* (últimos 3 anos)	Mamografia^†^ (últimos 2 anos)	Glicemia (últimos 3 anos)	Colesterol (últimos 3 anos)	Pressão arterial (último ano)
Geral					
ICC	0,15	0,12	0,18	0,22	0,19
	(0,11-0,21)	(0,08-0,18)	(0,14-0,24)	(0,17-0,29)	(0,13-0,28)
Mediana OR	2,07	1,91	2,28	2,54	2,33
	(1,76-2,38)	(1,64-2,19)	(1,99-2,57)	(2,14-2,93)	(1,84-2,81)
Sem plano de saúde					
ICC	0,17	0,14	0,16	0,21	0,24
	(0,12-0,24)	(0,09-0,21)	(0,12-0,22)	(0,15-0,28)	(0,16-0,35)
Mediana OR	2,22	2,02	2,14	2,41	2,65
	(1,86-2,56)	(1,69-2,35)	(1,84-2,44)	(2,00-2,82)	(1,99-3,31)
Afiliação a plano privado de saúde					
ICC	0,38	0,32	0,43	0,43	0,40
	(0,21-0,58)	(0,20-0,47)	(0,28-0,60)	(0,24-0,64)	(0,21-0,63)
Mediana OR	3,83	3,27	4,56	4,46	4,15
	(1,72-5,93)	(2,02-4,52)	(2,27-6,85)	(1,63-7,28)	(1,36-6,93)

Fonte: Elaboração própria, com base em dados do ELSI-Brasil.

ICC: coeficiente de correlação intraclasse; MOR: mediana da odds ratio.

*50 a 64 anos; ^†^50 a 69 anos.

A Tabela 3 apresenta os resultados das associações bruta e ajustada por potenciais fatores de confusão entre o IBP e os desfechos, estratificada pela afiliação a plano privado de saúde.

**Tabela 3.** Associação (odds ratio [intervalo de confiança de 95%]) entre Índice Brasileiro de Privação e uso de serviços de saúde entre mulheres mais velhas (ELSI Brasil, 2015-2016).

Modelo	Papanicolau* (até 3 anos)	Mamografia^†^ (até 2 anos)	Glicemia (até 3 anos)	Colesterol (até 3 anos)	Pressão arterial (último ano)
Com plano de saúde					
Não ajustado	0,85	0,99	**0,79**	**0,73**	0,84
	(0,73-1,00)	(0,86-1,13)	**(0,68-0,92)**	**(0,63-0,86)**	(0,68-1,04)
Ajustado^‡^	0,87	1,02	**0,82**	**0,71**	0,89
	(0,73-1,03)	(0,87-1,21)	**(0,69-0,96)**	**(0,59-0,87)**	(0,70-1,12)
Sem plano de saúde					
Não ajustado	0,97	0,99	**0,89**	**0,88**	1,03
	(0,91-1,02)	(0,94-1,04)	**(0,86-0,93)**	**(0,84-0,93)**	(0,96-1,09)
Ajustado^‡^	0,97	0,98	**0,91**	**0,89**	1,05
	(0,91-1,03)	(0,92-1,03)	**(0,87-0,96)**	**(0,85-0,95)**	(0,98-1,13)

Fonte: Elaboração própria, com base em dados do ELSI-Brasil.

*50 a 64 anos; ^†^50 a 69 anos; ^‡^Estimativas ajustadas por faixa etária, cor autorreferida, morar só e número de doenças crônicas (autorrelato de diagnóstico médico para as seguintes doenças: hipertensão arterial, diabetes, doença cardíaca, acidente vascular cerebral, doença pulmonar obstrutiva crônica, artrite ou reumatismo, depressão, câncer, insuficiência renal crônica).

Os valores em negrito representam associações estatisticamente significativas (p<0,05).

Nas análises ajustadas, observou-se, para mulheres com e sem plano de saúde, respectivamente, associação significativa entre o IBP e a realização dos exames de glicemia (odds ratio - OR 0,82; intervalo de confiança de 95% - IC95% 0,69-0,96 e OR 0,91; IC95% 0,87-0,96) e colesterol (OR 0,71; IC95% 0,59-0,87 e OR 0,89; IC95% 0,85-0,95) nos últimos três anos, sendo maior a chance de realização desses exames pelas mulheres residentes nos setores censitários de menor privação.

Os ajustes não modificaram de forma relevante os achados encontrados. Não houve associação significativa entre IBP e a realização de Papanicolau, mamografia e aferição da pressão arterial, independentemente da fonte de atenção. A Figura 2 ilustra esses resultados por meio das estimativas das probabilidades preditas de realização dos exames segundo o IBP e a afiliação a plano privado de saúde, ajustadas por faixa etária, cor autorreferida, arranjo domiciliar e número de doenças crônicas.

**Figura 2.** Probabilidade predita^‡^ de realização dos exames preventivos de saúde segundo o Índice Brasileiro de Privação e fonte de atenção à saúde, entre mulheres mais velhas (ELSI Brasil, 2015-2016).

IBP: Índice Brasileiro de Privação.

*50 a 64 anos; ^†^50 a 69 anos; ^‡^Ajustadas por faixa etária, cor autorreferida, morar só e número de doenças crônicas.

Fonte: Elaboração própria, com base em dados do ELSI-Brasil.

## DISCUSSÃO

Este estudo investigou a existência de iniquidades no uso de serviços de saúde entre mulheres com 50 anos e mais, especificamente na realização dos exames de mamografia e Papanicolau, dosagem de colesterol e glicemia e aferição da pressão arterial. Como principais resultados, destacamos a menor realização de exames de glicemia e colesterol por mulheres que residem em setores censitários de maior vulnerabilidade socioeconômica, independentemente da afiliação a plano privado de saúde.

É sabido que indivíduos que possuem plano privado de saúde realizam mais consultas médicas, com menor tempo de espera para conseguir consultas, além de maior facilidade para acessar os especialistas^35^. Estudos brasileiros demonstram que mulheres com plano de saúde realizam mais mamografias e Papanicolau^10^ ^,^ ^13^. Tendência similar ocorre nos EUA, onde a ausência de plano de saúde reduziu a realização desses exames em 31 e 41%, respectivamente, além de impactar a dosagem de colesterol (24% menor)^36^. Na Colômbia, também se observa redução da probabilidade de realizar mamografia e dosagem de glicemia. Já no México, a posse de plano favorece a realização de exames de colesterol, glicemia e aferição da pressão arterial^37^ ^,^ ^38^.

Para além dos fatores individuais, diversos estudos mostram que o contexto socioeconômico, comumente avaliado por índices como IDH e Gini, é um determinante da utilização de serviços de saúde^19^ ^,^ ^22^.

Neste trabalho, a caracterização do contexto socioeconômico baseou-se no IBP, mensurado em nível de setor censitário^29^, um diferencial comparado a outros índices. As análises de associação entre o IBP e os indicadores de uso de serviços de saúde por brasileiras mais velhas mostraram maior chance de realização dos exames de colesterol e glicemia entre as residentes em áreas de menor privação, independentemente das características individuais e da fonte de atenção. Estudos que utilizam medidas socioeconômicas contextuais no nível de setor censitário em análises de desigualdade são escassos na literatura. Por outro lado, estudos que avaliaram a associação entre IDH e Gini com a realização de mamografia e Papanicolau revelaram que o IDH das capitais esteve positivamente associado à maior realização dos exames. Além disso, demonstraram que maior desigualdade de renda, mensurada pelo índice de Gini, e menor IDH se associaram a dificuldades no acesso e menor prevalência na realização desses exames^13^ ^,^ ^21^. Evidências internacionais reforçam a associação entre o contexto socioeconômico e a utilização de serviços de saúde por mulheres. No Texas, mulheres residentes em municípios mais pobres realizaram menos o exame preventivo de Papanicolau^39^. Já em Ontário, no Canadá, residentes de bairros com menor quintil de renda realizaram menos exames de mamografia, dosagem de colesterol e glicemia^40^.

No presente estudo, a realização do Papanicolau, da mamografia e a aferição da pressão arterial não apresentaram associação significativa com o IBP, mostrando que a realização desses exames não é determinada pelo nível de privação do setor censitário, evidência contrária à observada para as dosagens de glicemia e de colesterol. Apesar de controvérsias na literatura sobre a efetividade de campanhas, principalmente com relação ao alcance do público-alvo^41^ ^,^ ^42^ ^,^ ^43^, uma hipótese levantada para este resultado é a eficácia das campanhas direcionadas para a conscientização da importância da realização de mamografia e Papanicolau, já bem estabelecidas e bastante difundidas, e que podem estar atuando como mitigadores do efeito socioeconômico contextual na realização dos exames. Já a aferição da pressão arterial, muito frequente para toda a população, pode ser realizada em qualquer consulta médica^44^, além de estar disponível em diferentes pontos de atenção e estabelecimentos, como farmácias privadas que ofertam essa ação, além da possibilidade de compra dos aparelhos. Por outro lado, estratégias semelhantes não são observadas para a dosagem de glicemia e de colesterol, o que torna a realização desses exames mais dependente de fatores socioeconômicos contextuais, que podem atuar como facilitadores ou barreiras de acesso.

A utilização do IBP como variável de caracterização socioeconômica contextual do setor censitário de residência possibilitou avaliar a associação entre o ambiente socioeconômico proximal das mulheres e a utilização dos serviços de saúde. Ao contrário das análises contextuais baseadas em áreas geográficas de maior abrangência^19^ ^,^ ^23^ ^,^ ^45^ e, portanto, com maior heterogeneidade interna, como municípios e Unidades da Federação, as evidências aqui encontradas colaboram para auxiliar os gestores na identificação de áreas com menor probabilidade de realização desses exames pelas mulheres residentes e, consequentemente, na implementação de ações e estratégias mais assertivas.

Este trabalho traz contribuições para a literatura. Trata-se de estudo realizado com amostra representativa da população brasileira de 50 anos e mais não institucionalizada, residente nas áreas urbanas e rurais dos municípios de pequeno, médio e grande porte. A análise dos dados foi feita com base em metodologia estatística adequada, utilizando o modelo multinível, e contou com o emprego de índice criado para avaliar o nível socioeconômico contextual do setor censitário.

Por outro lado, uma limitação importante diz respeito ao viés de informação, pois os desfechos foram autorrelatados e pessoas afetadas por um problema ou alguma doença tendem a recordar mais a realização desses exames do que as não afetadas. Além disso, por seu delineamento transversal, não se pode estabelecer relação de causalidade entre as variáveis. Vale destacar que a mensuração do IBP foi feita com os dados do Censo 2010, desatualizados em relação à condição socioeconômica da população brasileira.

Em suma, os resultados mostraram menor realização de exames de glicemia e colesterol entre mulheres brasileiras mais velhas residentes em áreas de maior privação socioeconômica. Esses achados sublinham a necessidade de ações e políticas públicas voltadas para mulheres brasileiras mais velhas que têm o serviço público como principal fonte de atenção à saúde e residem em setores censitários de maior privação, como estratégia para reduzir as iniquidades na utilização dos serviços de saúde. Ademais, os achados reforçam a importância de ampliar o escopo das políticas de saúde da mulher para além do rastreamento de cânceres ginecológicos, institucionalizando a avaliação regular de exames, como glicemia e colesterol, garantindo uma assistência integral que considere também os determinantes sociais de saúde.
